# Glial Cell Ceruloplasmin and Hepcidin Differentially Regulate Iron Efflux from Brain Microvascular Endothelial Cells

**DOI:** 10.1371/journal.pone.0089003

**Published:** 2014-02-12

**Authors:** Ryan C. McCarthy, Daniel J. Kosman

**Affiliations:** Department of Biochemistry, University at Buffalo, School of Medicine and Biomedical Scienes, Buffalo, New York, United States of America; Auburn University, United States of America

## Abstract

We have used an *in vitro* model system to probe the iron transport pathway across the brain microvascular endothelial cells (BMVEC) of the blood-brain barrier (BBB). This model consists of human BMVEC (hBMVEC) and C6 glioma cells (as an astrocytic cell line) grown in a transwell, a cell culture system commonly used to quantify metabolite flux across a cell-derived barrier. We found that iron efflux from hBMVEC through the ferrous iron permease ferroportin (Fpn) was stimulated by secretion of the soluble form of the multi-copper ferroxidase, ceruloplasmin (sCp) from the co-cultured C6 cells. Reciprocally, expression of sCp mRNA in the C6 cells was increased by neighboring hBMVEC. In addition, data indicate that C6 cell-secreted hepcidin stimulates internalization of hBMVEC Fpn but only when the end-feet projections characteristic of this glia-derived cell line are proximal to the endothelial cells. This hepcidin-dependent loss of Fpn correlated with knock-down of iron efflux from the hBMVEC; this result was consistent with the mechanism by which hepcidin regulates iron efflux in mammalian cells. In summary, the data support a model of iron trafficking across the BBB in which the capillary endothelium induce the underlying astrocytes to produce the ferroxidase activity needed to support Fpn-mediated iron efflux. Reciprocally, astrocyte proximity modulates the effective concentration of hepcidin at the endothelial cell membrane and thus the surface expression of hBMVEC Fpn. These results are independent of the source of hBMVEC iron (transferrin or non-transferrin bound) indicating that the model developed here is broadly applicable to brain iron homeostasis.

## Introduction

Dysregulation of iron homeostasis has been associated with a variety of neurodegenerative disorders. Thus, as the major user of metabolic energy (on a per-weight basis) the central nervous system (CNS) strongly relies on iron while at the same time is highly vulnerable to iron-induced oxidative stress. Indeed, progressive accumulation of iron in a normal aging brain [1] or pathologic alterations of its homeostasis can be the cause of or contribute to the cellular degeneration observed in many neurologic disorders [1]–[4]. In addition, a disruption of iron handling likely plays an important role in acute neuronal injury characterized by an increase in intracellular free iron; cerebral ischemia is an example of such an injury state [5].

The primary regulator of brain iron is the layer of brain microvascular endothelial cells (BMVEC) which, together with underlying astrocytes form the blood-brain barrier (BBB). BMVEC lack the fenestrations common to the endothelial cells in peripheral capillaries; in contrast, they form tight-junctions and thus regulate the transport of polar molecules across the BBB [6], [7]. In this report we provide experimental evidence in support of the mechanism by which the iron accumulated by BMVEC is exported from the basal (brain; abluminal) surface of these cells, thus trafficking plasma iron across the BBB and into the brain interstitium.

We based our experimental design on the relative spatial proximity of astrocytes to BMVEC during different periods of neonatal development. During embryogenesis, astrocytes are underdeveloped and spatially absent from the local microenvironment surrounding the basal surface of the BVMEC [8]. From approximately postnatal day 0–14 (P0-P14) astrocytes are extending their endfeet into the local microenvironment surrounding the basal surface of BMVEC [8]. Ensheathment of BMVEC by astrocytes begins with postnatal ontogenesis and is essentially complete by the start of post-natal week three (P14) [8]; with respect to iron trafficking, a depletion in BMVEC hephaestin (Hp) and ferroportin (Fpn) has been demonstrated at this developmental juncture [9]. With the lack of fluid circulation in the brain parenchyma we speculate that proximal cell-cell communication between BMVEC and their spatially adjacent astrocytes modulates the regulation of brain iron uptake during development.

Brain iron uptake from serum requires importation of that iron into BMVEC with the subsequent export of that iron at the basal surface. BMVEC iron is exported through the ferrous iron permease Fpn in conjunction with an exocytoplasmic ferroxidase such as Hp or ceruloplasmin (Cp) [10]. Both Fpn and Hp have been identified in the BMVEC of the BBB [9]–[13]. In addition, soluble Cp (sCp), ferritin heavy chain (FHC) and amyloid-β precursor protein (APP) have ferroxidase activity as well and thus may play a role in cellular iron efflux [14], [15], although in the case of APP this remains controversial [16], [17]. The astrocyte secretome contains both sCp and FHC, and APP is expressed by both BMVEC and astrocytes [18]–[21].

While astrocytes express proteins that enhance Fpn activity, they also express hepcidin, a peptide hormone which induces the turnover of Fpn. Examination of the murine CNS has revealed that glial fibrillary acidic protein-positive cells (astrocytes) express hepcidin [22]. Hepcidin binds to and induces ubiquitination of Fpn; this triggers Fpn internalization and degradation [23]–[25]. We propose that astrocytes regulate the flux of iron from BMVEC through the secretion of hepcidin. Evidence for this mechanism comes from hepcidin-knockout mice (Hepc^−/−^) that display increased Fpn immunoreactivity in the endothelial cells of the blood-retinal barrier; this increased Fpn results in an increased iron accumulation in the retina relative to that observed in retinas of wild type mice [26]. Plasma membrane expression of Fpn in and iron export from BMVEC in these animals has not been investigated.

Here, we provide new insight in regards to the regulation by C6 cells of iron efflux from a human brain microvasculature cell line (hBMVEC). First, using an *in vitro* model BBB system, we show that this glia-derived cell line stimulates hBMVEC iron efflux *via* a paracrine mechanism involving secreted, diffusible ferroxidase-active proteins. Second, we demonstrate that sCp, which is a component of the astrocyte secretome [18], is secreted by C6 cells and stimulates hBMVEC iron efflux. Furthermore, we demonstrate that the expression of sCp in C6 cells is up-regulated by a factor secreted by hBMVEC. Lastly, we provide evidence that C6 cell proximity dictates the internalization of hBMVEC Fpn likely *via* secretion of the peptide hormone hepcidin. Based on our results, we propose a model in which astrocyte-expressed proteins both up- and down-regulate BMVEC iron efflux as a result of physiologically essential regulatory interactions between the BMVEC and astrocytes of the BBB.

## Results

### C6 cell proximity modulates hBMVEC iron efflux in an *in vitro* blood-brain barrier

**Figure 1:**
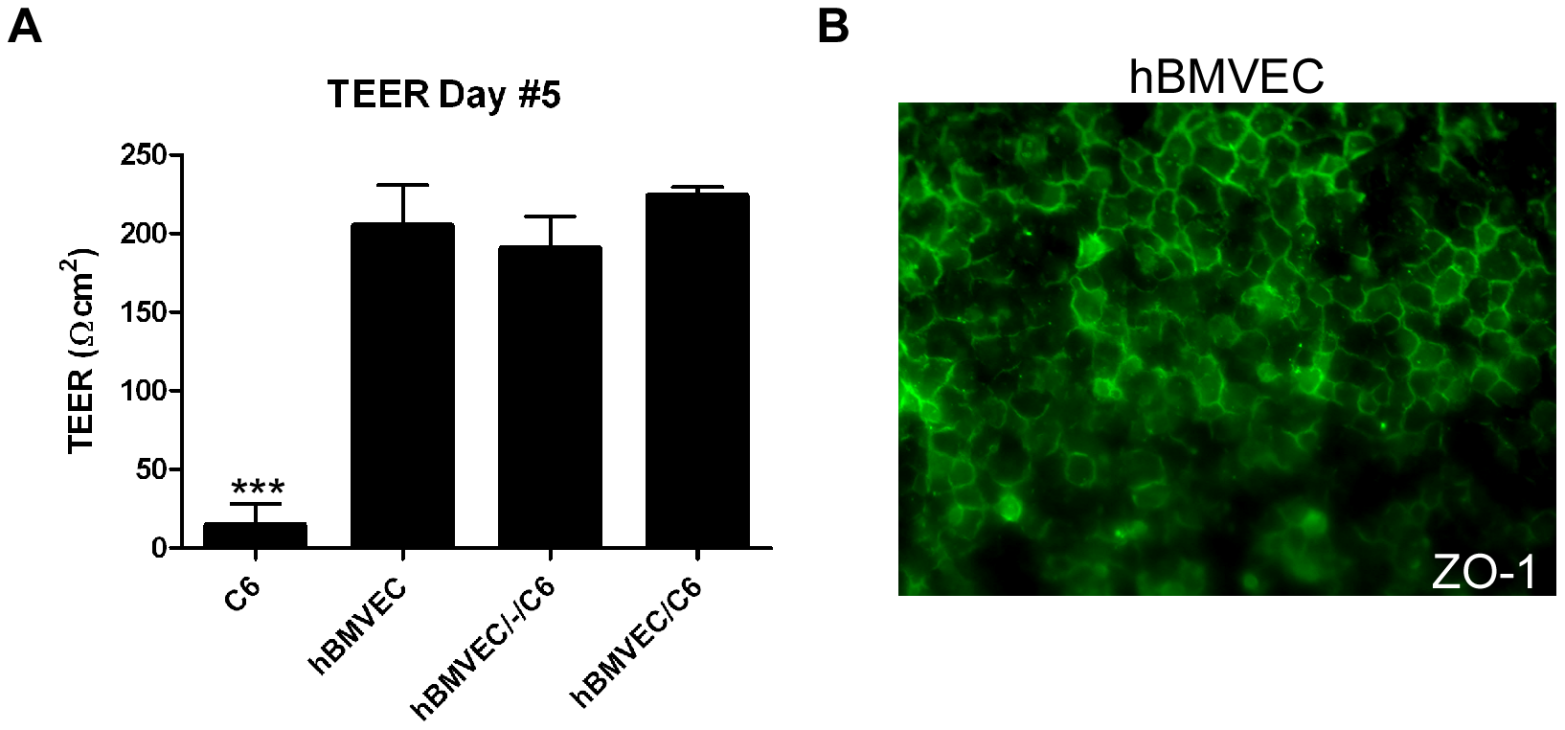
Establishing an *in vitro* model BBB system. (A) Transendothelial electrical resistance (TEER) values for hBMVEC grown alone, hBMVEC grown distal (hBMVEC/-/C6), or proximal to C6 glioma cells (hBMVEC/C6) in a transwell model system for 5 days. In each case, hBMVEC were grown on the upper-surface of the transwell membrane; when present, C6 cells were grown on the bottom of the lower chamber (distal) or on the undersurface of the transwell membrane (proximal). One-way ANOVA analysis was conducted: ***P-value<0.0001. Data are represented as means ± S.D. (n = 3–6, experimental replicates). (B) Indirect immunofluorescence image of hBMVEC zona occludens 1 (ZO-1) in hBMVEC grown alone on the upper-surface of the transwell membrane.

BMVEC are polarized; their apical (luminal) surface faces the blood and their basal (abluminal) surface faces the brain interstitial fluid. To replicate this orientation *in vitro* we utilized a transwell system [27]. The upper chambers of the transwells were filled with media containing serum to mirror the luminal surface of the hBMVEC capillary milieu while the lower chamber contained media without serum to mirror the abluminal (brain) context. hBMVEC grown on the upper-surface of the transwell membrane formed tight-junctions after 5 days as demonstrated quantitatively *via* transendothelial electrical resistance (TEER). Equivalent resistance was observed when C6 cells were co-cultured on the bottom of the lower chamber (distal orientation) or on the undersurface of the transwell membrane (proximal orientation) (Fig. 1A). The presence of the tight-junction protein zona occludens 1 (ZO-1) was also observed in these cultures; the ZO-1 immunofluorescence in hBMVEC seeded alone is shown in Fig. 1B. Such data validate this model BBB [28], [29].

**Figure 2:**
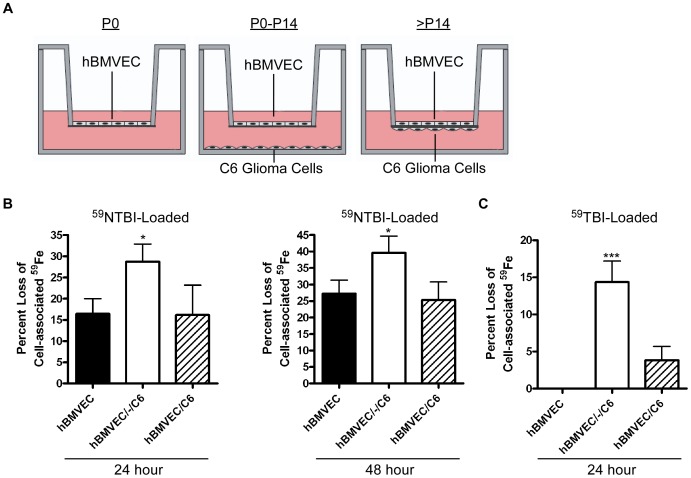
hBMVEC iron efflux is regulated by astrocyte proximity. (A) BBB developmental time points were mimicked *in vitro* in a transwell culture system. In the following experiments, hBMVEC were seeded either alone (filled bars; representative of rat postnatal day 0 (P0)), distal to C6 glioma cells (open bars; representative of rat postnatal days 0–14 (P0–P14)), or proximal to C6 glioma cells (hashed bars; representative of rat postnatal day 14 and beyond (>P14)). RPMI 1640 with serum (upper chamber) or without serum (lower chamber) was used. Except where noted, hBMVEC were loaded with ^59^Fe^II^-citrate (^59^NTBI) for 24 h after which point ^59^Fe efflux assays were performed. (B) The percent loss of hBMVEC-associated ^59^Fe quantified after loading (t = 0) was monitored at 24 h and 48 h as indicated. (C) hBMVEC were loaded with ^59^Fe-transferrin (^59^TBI) plus citrate for 24 h after which point ^59^Fe efflux assays were performed. The percent loss of hBMVEC-associated ^59^Fe relative to t = 0 was monitored at 24 h. One-way ANOVA statistical analyses were used to determine significance of the data at each time point. *P<0.05, ***P<0.001. Data are represented as means ± S.D. (n = 3–6, experimental replicates).

^59^Fe-efflux assays were performed to mimic the known patterns of hBMVEC ^59^Fe-flux at three points in development: 1) embryogenesis (P0); 2) postnatal ontogenesis (P0-P14); and 3) adulthood (>P14). To model these developmental periods *in vitro*, hBMVEC were seeded either alone (P0), with C6 glioma cells on the bottom of the lower chamber (distal) (P0-P14), or with C6 cells in contact on the undersurface of the transwell membrane (proximal) (>P14) (see illustration, Fig. 2A). C6 cells are commonly used as an astrocytic model in co-cultures with either BMVEC or neurons [30]–[32].

In all cases ^59^Fe was loaded into the upper chamber for 24 h, the cells were washed, and hBMVEC ^59^Fe-efflux was monitored subsequently at 0 h, 24 h and 48 h. The tightness of the barrier formation with respect to ^59^Fe in each orientation was nearly identical as indicated by the lack of ^59^Fe-leakage from the upper chamber into the lower chamber during loading (Fig. S1). Data are presented as the percentage of ^59^Fe lost from hBMVEC relative to the total cell-associated ^59^Fe at the beginning of the quantification of efflux (Figs. 2B and 2C). A significant increase in hBMVEC ^59^Fe-efflux occurred when C6 cells were grown distal to hBMVEC. In contrast, ^59^Fe-efflux was at a minimum when hBMVEC were grown alone or grown proximal to, and likely in contact with, the glial cell line (Figs. 2B and 2C). hBMVEC loaded with either ^59^Fe-citrate (^59^NTBI) or ^59^Fe-transferrin (^59^TBI) demonstrated a similar response to astrocyte proximity in regards to the pattern of ^59^Fe-efflux over time (Figs. 2B and 2C). Our data suggest that astrocytes enhance hBMVEC iron efflux when the two cell types are distal from one another suggesting that this stimulation could be due to a secreted, diffusible factor expressed by the glial cell line. Significantly, when the cells are grown proximally, this apparent stimulation of iron efflux is suppressed.

**Figure 3:**
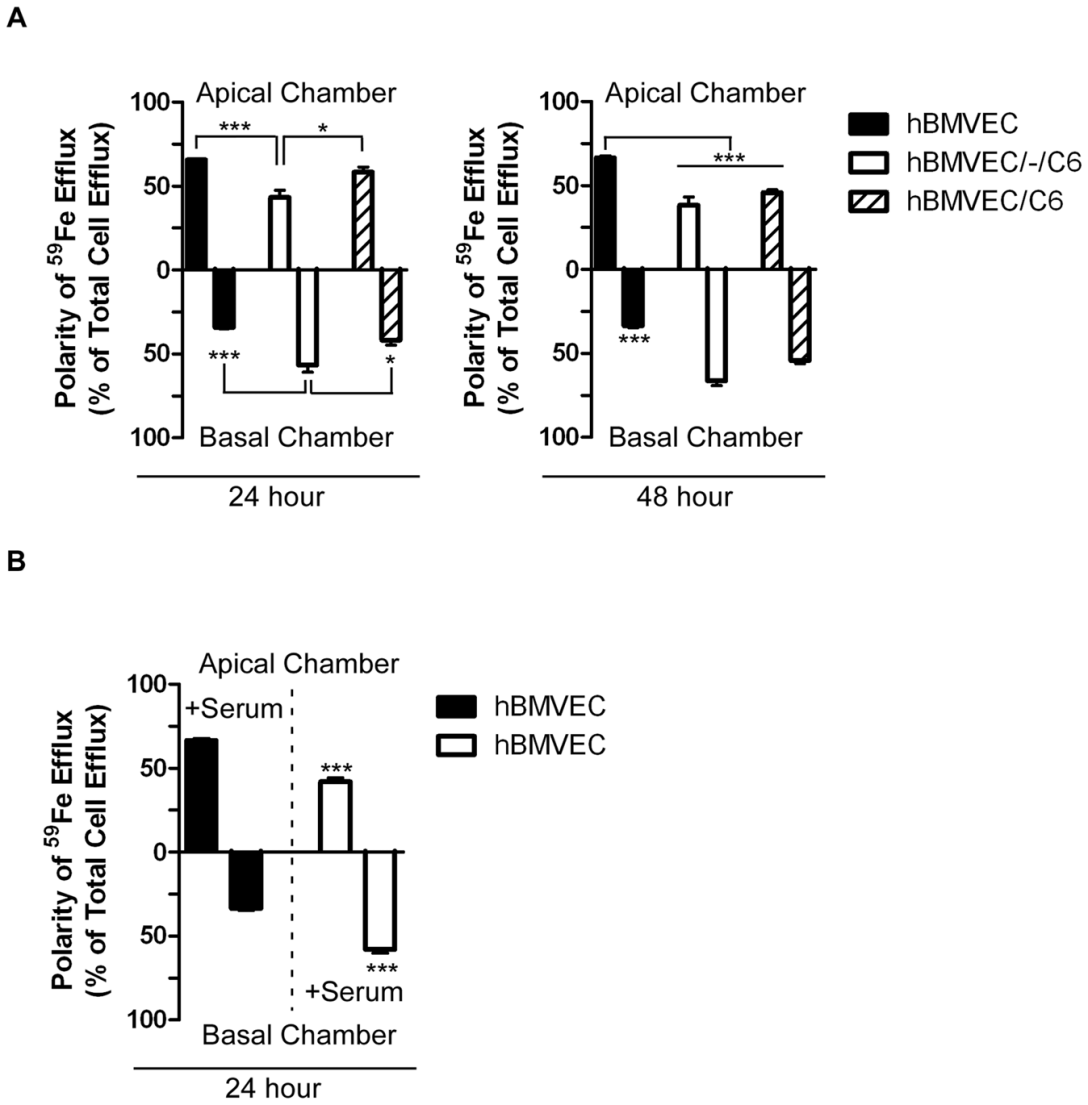
C6 glioma cells and serum influence the polarity of hBMVEC iron efflux. (A) hBMVEC were seeded either alone (hBMVEC), distal to (hBMVEC/-/C6), or proximal to C6 glioma cells (hBMVEC/C6) and were loaded for 24 h with ^59^NTBI after which point ^59^Fe efflux was quantified. RPMI 1640 with serum (upper chamber) or without serum (lower chamber) was used in the efflux assay. The polarity of hBMVEC-associated ^59^Fe efflux into the upper versus lower chamber was monitored at 24 h and 48 h as indicated. (B) In this experiment hBMVEC were seeded alone in transwell with RPMI 1640 in both upper and lower chambers. The polarity of hBMVEC-associated ^59^Fe efflux into the upper versus lower chamber was monitored at 24 h with serum added to the upper chamber only (closed bars), or to the lower chamber only (open bars). One-way ANOVA statistical analyses were used to determine significance of the data at each time point. *P<0.05, ***P<0.001. Data are represented as means ± S.D. (n = 3–6, experimental replicates).

Our transwell model BBB system allowed for quantification of apical *versus* basal hBMVEC iron efflux. Such a polarized efflux of, for example, cytokines from BMVEC has been quantified in this type of transwell model system [33]. In the absence of C6 cells, hBMVEC iron efflux was predominantly directed towards the upper chamber which contained serum (Fig. 3A). In contrast, the efflux of iron from hBMVEC was focused towards the lower chamber when C6 cells were co-cultured in either a distal or proximal orientation (Fig. 3A). By reversing the orientation of the serum (upper chamber –serum, lower chamber +serum), and in the absence of the glia-derived cells, hBMVEC iron efflux into the lower chamber was significantly enhanced (Fig. 3B, open bars). These data suggest the hypothesis that serum and C6 cell conditioned media (CCM) contain a factor(s) that modulates the polarity of iron flux from hBMVEC.

### Ferroxidase-active proteins in the astrocyte secretome and C6 cell-conditioned media stimulate hBMVEC iron efflux

We investigated what diffusible agent(s) secreted by C6 cells could potentiate the enhanced hBMVEC iron efflux observed in the distal orientation. Components of the astrocyte secretome have been identified, including those which possess ferroxidase activity [18], [34]. These include sCp, and ferritin heavy and light chains (FHC and FLC, respectively) all of which are found in serum as well. Note that the expression and secretion of sCp by astrocytes has been underappreciated [18], [35], [36]. We screened the ability of these proteins to stimulate hBMVEC ^59^Fe-efflux utilizing hBMVEC grown in monolayers; the monolayer system allowed for high-throughput analyses. In the absence of serum, hBMVEC monolayers loaded with ^59^Fe-citrate (NTBI) exhibited little ^59^Fe-efflux over 48 h (Table 1, left column).

**Table 1:** Ferroxidase activity, HepG2-, C6- and CG4-conditioned media, but not iron chelators stimulate hBMVEC iron efflux.

	% loss of hBMVEC-associated ^59^Fe Duration of efflux			% loss of hBMVEC-associated ^59^Fe Duration of efflux	
Media with Addition	t = 24 h	t = 48 h	Media Conditioned by:	t = 24 h	t = 48 h
**Control Media (RPMI)**	3±6	15±5	**RPMI** (control)	3±6	15±5
250 µM Citrate	14±5	19±6	C6 Glioma	32±7***	39±8****
10 µM Apo-Tf	12±9	18±12	Primary rat astrocytes	20±6**	30±5**
6.6 nM sCp	22±6**	26±9**	HepG2	25±5**	30±7**
1 µM H-Ferritin	14±5*	27±7*	Caco-2	10±4	6±7
5 µM H-Ferritin	28±5**	46±3****	HEK293T	10±4	23±5
1 µM L-Ferritin	17±8*	22±12	**RPMI** (^59^TBI-loaded)	5±7	5±5
5 µM L-Ferritin	20±6*	26±8*	C6 Glioma (^59^TBI-loaded)	26±1*	34±8**
0.5 µM FD1 Peptide	19±4*	31±4***	**Neural Basal Media**	9±7	8±9
6.6 nM sCp, 5 µM H-Ferritin, 0.5 µM FD1	27±5***	47±3****	Primary rat hippocampal neurons	9±5	9±6
			**M41 Media**	15±11	16±10
			CG4	17±6	18±13

Media in bold was supplemented with components listed or were conditioned for 24 h with the cell type as indicated; “Control” media was RPMI1640. Neural basal and M41 media are defined in “Materials and Methods”. Cells were loaded with ^59^Fe-Transferrin (^59^TBI) instead of ^59^Fe-citrate in one experiment as indicated. The FD1 peptide sequence: was HRERMSQVMREWEEAERQAKNL. CG4 is a bipotential oligodendroglial precursor cell line. A series of paired t-tests were used to analyze the data at each time point for each condition in comparison to Control Media.

*P<0.05,

**P<0.01,

***P<0.001,

****P<0.0001. Data are represented as means ± S.D. (n = 4–8; experimental replicates).

Indeed, in our monolayer system, components of the astrocyte secretome with ferroxidase activity (sCp, FHC, FLC), stimulated hBMVEC ^59^Fe-efflux; these data are presented in Table 1 (left column). Media conditioned by C6 cells or by primary rat astrocytes had an similar stimulatory effect; in the case of glial cell conditioned media, a similar stimulation of efflux was quantified with hBMVEC loaded with ^59^Fe-transferrin (^59^TBI, Table 1, right column). Conditioned media from HepG2 cells, which secrete sCp and FHC [34], also potentiated hBMVEC ^59^Fe-efflux. In contrast, media conditioned by HEK293T, Caco-2 cells, rat hippocampal neurons or CG4 cells (an undifferentiated glial progenitor cell line) failed to stimulate such efflux (Table 1, right column). Also without effect on efflux were apo-transferrin and citrate, both of which contribute to mammalian iron trafficking.

A sequence within the E2 domain of APP (residues 402–417 in hAPP770) has recently been characterized as a ferroxidase [14], although this finding has been challenged [16], [17]. Since astrocytes express APP [20], [37] we hypothesized that the putative ferroxidase-active cleavage product of APP could stimulate hBMVEC ^59^Fe-efflux. We used a 22 amino acid synthetic peptide, FD1, homologous to the E2 domain of APP that has been characterized previously as stimulating iron-efflux activity [14]. Indeed, this peptide stimulated hBMVEC ^59^Fe-efflux (Table 1, left column) suggesting that APP has the capacity to catalyze BMVEC iron efflux into the brain. The non-additive effects of sCp, FHC, and FD1 on the stimulation of hBMVEC ^59^Fe-efflux suggest that these astrocyte-secreted species stimulate hBMVEC iron efflux *via* an overlapping pathway and thus are functionally redundant in this respect.

**Figure 4:**
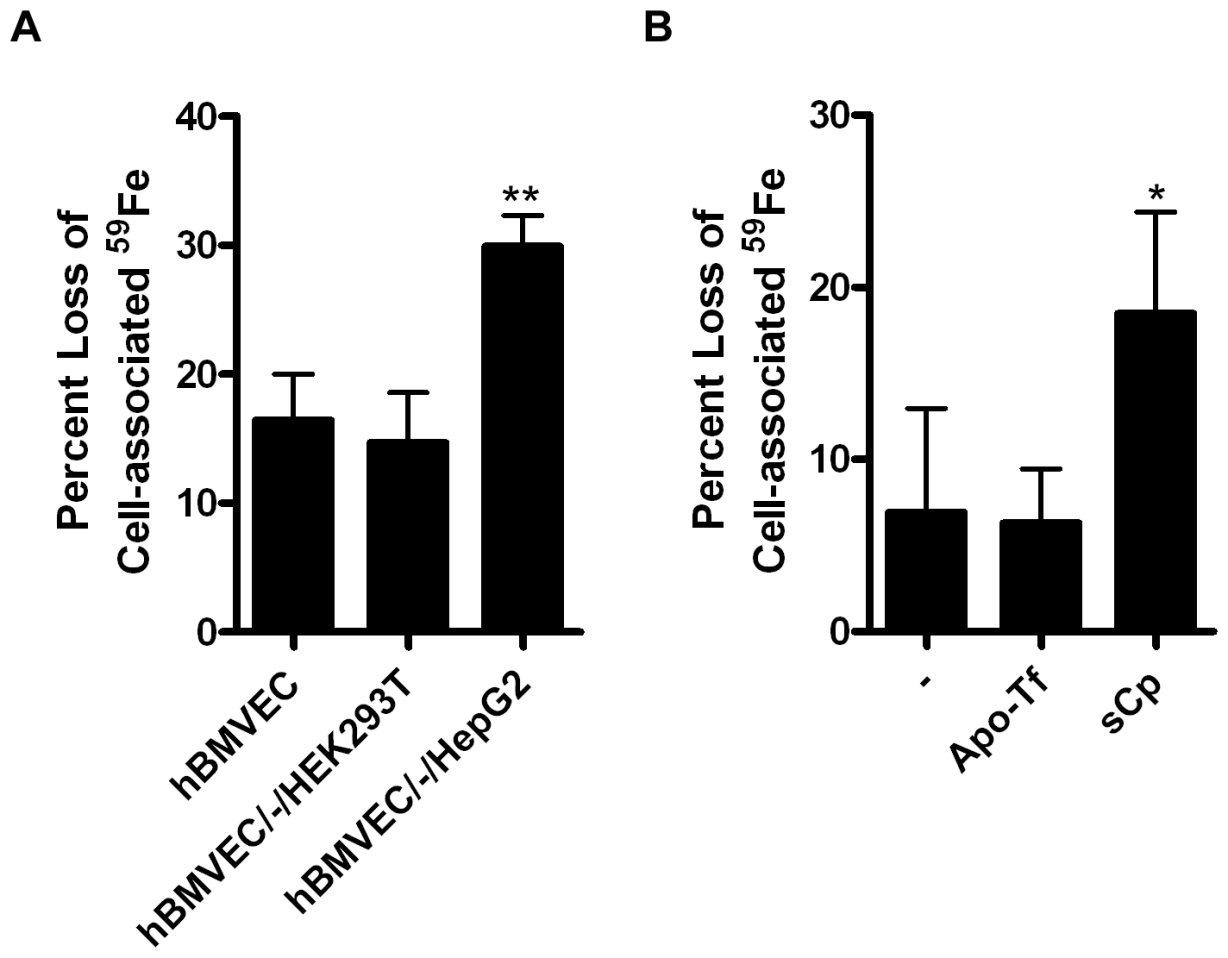
Media containing ferroxidase active proteins enhance hBMVEC iron efflux in a model BBB system. (A) hBMVEC were seeded in transwell either alone (hBMVEC), distal to HEK293T cells (hBMVEC/-/HEK293T), or distal to HepG2 cells (hBMVEC/-/HepG2). hBMVEC were loaded with ^59^Fe^II^-citrate for 24 h after which point ^59^Fe efflux assays were performed. The percent loss of the initial hBMVEC-associated ^59^Fe was quantified at 24 h (n = 4-6, experimental replicates). (B) ^59^Fe efflux assays were performed on hBMVEC seeded alone in transwell with the addition of either apo-Tf (10 µM) or sCp (6.6 nM) to the lower chamber during efflux. The percent loss of hBMVEC-associated ^59^Fe was quantified at 24 h. One-way ANOVA statistical analyses were used to determine significance. *P<0.05, **P<0.005. Data are represented as means ± S.D. (n = 6–12, experimental replicates).

We tested the inference that sCp stimulated iron efflux in our transwell system as well. HepG2 cells, which secrete sCp, served as a positive control, while HEK293T cells, which do not, provided a negative control. One or the other cell line was seeded on the bottom of the lower chamber in our transwell system, distal to hBMVEC. hBMVEC iron efflux was stimulated by HepG2 but not by HEK293T cells grown in this orientation (Fig. 4A). Also, whereas addition of purified human sCp to the lower chamber of a transwell containing only hBMVEC enhanced iron efflux, apo-Tf did not (Fig. 4B). These data support the hypothesis that cells which secrete sCp can stimulate iron efflux from hBMVEC.

### C6 cell-induced hBMVEC iron efflux depends on C6 cell-secreted soluble ceruloplasmin

**Figure 5:**
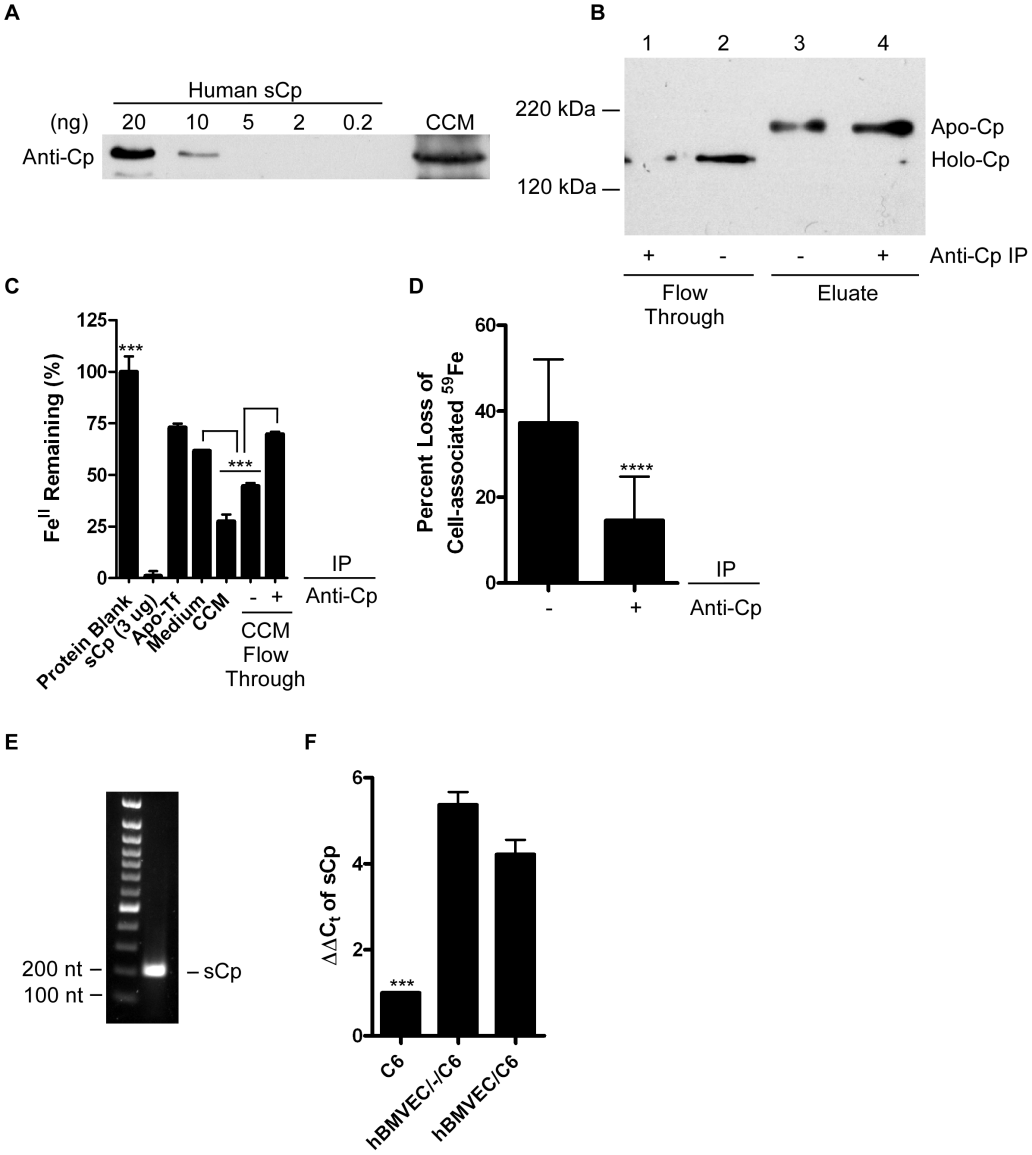
C6 cell sCp is a diffusible agent that supports hBMVEC iron efflux. (A) Immunoblot probing for sCp in C6 cell monolayer conditioned media (CCM). The protein band shown has estimated mass of 150 kDa. Titrations of human sCp are shown and analysis of their relative optical densities reveals the concentration of sCp in the CCM used in panels C and D to be ∼1 nM. (B) Immunoblot confirming the immunodepletion of sCp from CCM. Flow through and eluted samples of both the control (- anti-Cp, lanes 2 & 3) and the experimental (+ anti-Cp, lanes 1 & 4) samples are shown (Holo-Cp migrates as *M*_r_ ∼150 kDa; Apo-Cp migrates as *M*_r_ ∼200 kDa). (C) Ferroxidase assays were performed utilizing the colorimetric indicator ferrozine; the Fz:Fe^II^ complex exhibits absorbance at 550 nm (ε = 27.9 mM^−1^cm^−1^). Data are represented as the percentage of Fe^II^ remaining in solution relative to the no protein control. CCM was immunodepleted (+ Anti-Cp) or not (− Anti-Cp) of sCp (n = 3–6, technical replicates) as in panel B. sCp and apo-transferrin (Tf) were used as positive and negative ferroxidase controls, respectively. (D) hBMVEC ^59^Fe efflux assays were conducted in the presence of CCM immunodepleted of sCp (+ Anti-Cp) or not (− Anti-Cp). Iron efflux was monitored over 24 h. Data are represented as the percent loss of initial cell-associated ^59^Fe (n = 6, experimental replicates). (E) RT-PCR detection of sCp-specific mRNA in C6 cell total RNA. PCR product was resolved on a 1.7% agarose gel. (F) qPCR of sCp transcript abundance in total RNA isolated from C6 glioma cells seeded in transwell alone (C6 only), distal to (hBMVEC/-/C6), or proximal to hBMVEC (hBMVEC/C6). One-way ANOVA statistical analyses (C and F) or paired t-test (D) were used to determine significance. ***P<0.001, ****P<0.0001. Data are represented as means ± S.D.

Analysis by immunoblot of concentrated CCM from a C6 monolayer demonstrated the presence of sCp (Fig. 5A). A standard curve using purified human sCp in this western analysis provided an estimate of 1 nM for the concentration of sCp in this media. We performed a ferroxidase assay on the CCM utilizing the ferrous iron chelating agent ferrozine, which acts as a colorimetric indicator of the presence of Fe^II^ in solution. Soluble Cp was used as a positive control, while apo-Tf served as a negative control in this ferroxidase assay (Fig. 5C). The ferroxidase assay demonstrated that this activity in CCM was ∼2-fold greater than in unconditioned media (Fig. 5C). The CCM was cleared of sCp by immunodepletion (confirmed by western blot, Fig. 5B) and re-assayed for ferroxidase activity; note that the shift of sCp from holo-Cp to apo-Cp is expected as copper loss from sCp occurs due to the low-pH elution buffer. A significant loss of ferroxidase activity from the immune-depleted CCM (+anti-Cp) was observed (Fig. 5C). The non-immunodepleted CCM control (-anti-Cp) lost a smaller fraction of the original ferroxidase activity, an effect which could be attributed to the non-specific binding of sCp to the column as seen in the eluted sample (Fig. 5B, lane 3 and Fig. 5C). Importantly, this sCp-depleted CCM (+anti-Cp) stimulated significantly less hBMVEC iron efflux as compared to the non-immunodepleted control CCM (-anti-Cp) (Fig. 5D). These data support the hypothesis that C6 cell-secreted sCp supported the major fraction of CCM-induced hBMVEC iron efflux in our model system. We propose that the serum stimulation of efflux is due at least in part to sCp as well.

### hBMVEC paracrine signaling enhances C6 cell sCp gene expression

The data in Fig. 2 indicate that in this transwell culture system C6 cells enhance hBMVEC iron efflux when seeded in a distal but not a proximal orientation. There are two simple explanations for this reduction in hBMVEC iron efflux with the glial cell line in the proximal orientation: 1) a loss of C6 cell sCp expression; or 2) a loss of hBMVEC iron efflux function. We investigated the former of these two hypotheses by examining the effect of hBMVEC proximity on C6 cell sCp gene expression. Using RT-PCR, we confirmed the presence of sCp transcript in the C6 glioma cell line (Fig. 5E). To quantify the effect of hBMVEC proximity on C6 glioma sCp transcript expression, C6 cells were seeded in the lower chamber alone, distal to, or proximal to hBMVEC and allowed to grow for 5 days. Total RNA was collected from the glioma cells and quantified for sCp-specific mRNA using qPCR. Growth of hBMVEC in transwell with C6 cells in any orientation resulted in a 4–5-fold increase in C6 glioma sCp transcript abundance compared to C6 cells grown alone (Fig. 5F). These data suggest that the reduction of iron efflux from hBMVEC when grown proximal to astrocytes is not the result of a loss of astrocyte sCp expression.

### Glial cell proximity regulates the expression of hBMVEC Fpn

We have demonstrated that when C6 cells come into contact (proximal) with hBMVEC, their stimulatory effect on iron efflux from hBMVEC is inhibited. This *in vitro* observation correlates with the *in vivo* one, namely that iron uptake into the brain and BMVEC Fpn expression is diminished upon complete ensheathment of brain capillaries by astrocytes after P14 [8], [9]. Thus, we reasoned that the loss of hBMVEC iron efflux when hBMVEC and C6 cells were grown proximally on opposing surfaces of the transwell membrane was due to a loss of hBMVEC Fpn iron export function. We used our BBB model system to test this possibility.

**Figure 6:**
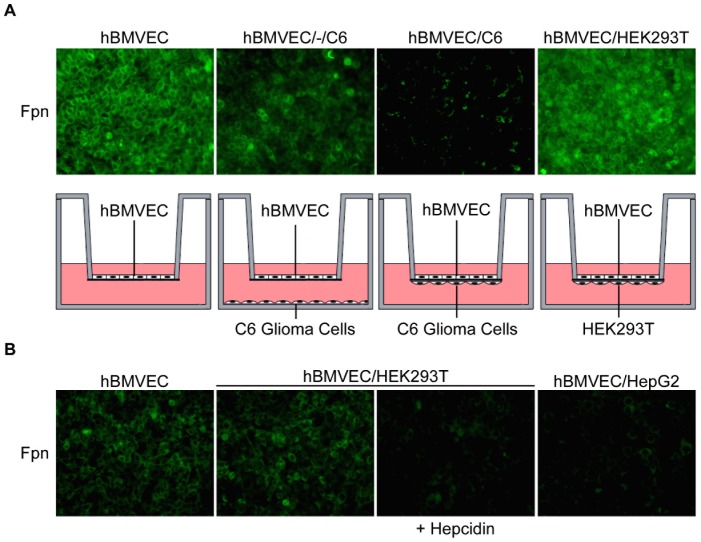
Internalization of hBMVEC Fpn correlates with presence of exogenous hepcidin. (A) Indirect immunofluorescence was used to assess hBMVEC Fpn expression when seeded in transwell either alone (hBMVEC), distal to C6 cells (hBMVEC/-/C6), proximal to C6 cells (hBMVEC/C6), or proximal to HEK293T cells (hBMVEC/HEK293T). (B) Indirect immunofluorescence was used to assess hBMVEC Fpn expression when seeded in transwell either alone (hBMVEC), or proximal to HEK293T cells (hBMVEC/HEK293T) in the presence or absence of exogenous hepcidin (200 nM; for 24 h). hBMVEC Fpn expression was also assessed when seeded proximal to HepG2 cells (hBMVEC/HepG2) in transwell. Images are at 20X magnification to demonstrate uniformity of response across the culture.

**Figure 7:**
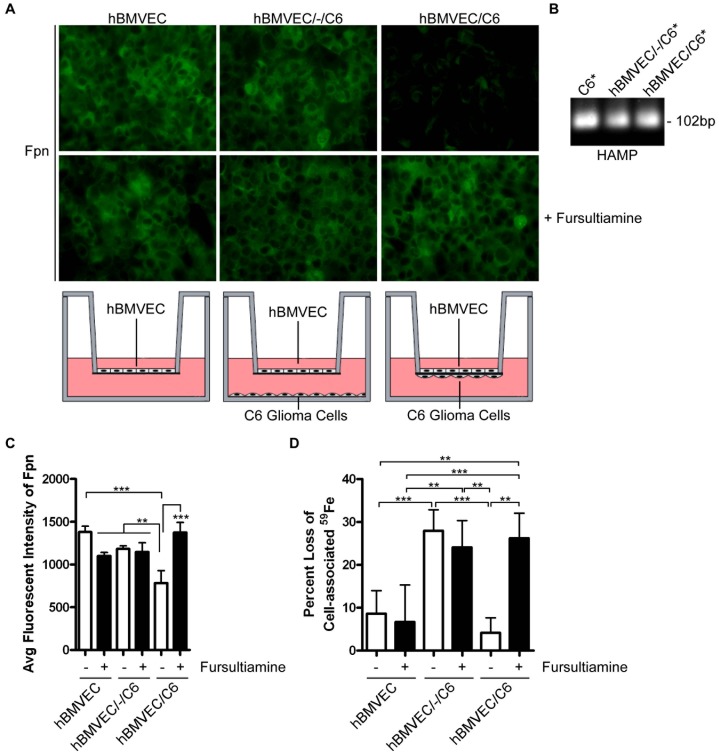
Astrocyte-secreted hepcidin induces hBMVEC Fpn internalization. (A) Indirect immunofluorescence probing for hBMVEC Fpn when hBMVEC are seeded in transwell either alone (hBMVEC), distal to (hBMVEC/-/C6), or proximal to (hBMVEC/C6) C6 glioma cells. Where indicated, fursultiamine (10 µM) was added for 24 h. The images were obtained with a 40X objective. (B) RT-PCR of HAMP (hepcidin transcript) in total RNA isolated from C6 cells seeded in transwell alone (C6), distal to hBMVEC (hBMVEC/-/C6), or proximal to hBMVEC (hBMVEC/C6). (C) Average fluorescent intensity of Fpn from 3–4 separate fields of view from each condition in (A) was obtained using the Zeiss AxioVision software. (D) The percent loss of initial hBMVEC-associated ^59^Fe in the presence or absence of 10 µM fursultiamine was monitored at 24 h (n = 6, experimental replicates). One-way ANOVA statistical analyses were used to determine significance. **P<0.01, ***P<0.001. Data are represented as means ± S.D.

Transwells were assembled with the appropriate cell types or media as indicated (Fig. 6A) and hBMVEC Fpn was examined by indirect immunofluorescence. Transwells with hBMVEC and C6 glioma cells on opposite sides of the membrane (proximal orientation) displayed a noticeable loss in hBMVEC Fpn as compared to hBMVEC alone or with C6 cells grown on the bottom of the lower compartment (distal orientation). As a negative control, HEK293T cells were seeded proximal to hBMVEC; no change in hBMVEC Fpn was observed in this co-culture combination (Fig. 6A). In contrast, HepG2 cells grown proximal to hBMVEC (hBMVEC/HepG2) did induce a loss in hBMVEC Fpn fluorescence (Fig. 6B). We then tested the possibility that hepcidin, which is expressed by both glial [38] and HepG2 cells [39] and which down-regulates Fpn [23], [40], induced the loss of hBMVEC Fpn. Indeed, addition of hepcidin (200 nM) to a hBMVEC/HEK293T co-culture (the negative control in panel A) resulted in an equivalent loss of Fpn as seen in hBMVEC cultured proximally to either C6 or HepG2 cells (Fig. 6B). The decrease in hBMVEC Fpn intensity when seeded proximal to C6 glioma cells was significant as determined by quantitation of the average fluorescent intensities of at least three separate fields of view (*cf* Fig. 7C, open bars). The decrease in hBMVEC Fpn abundance when the endothelial cells were grown proximal to C6 cells correlated with the suppression of ^59^Fe-efflux observed in this orientation (*cf* Fig. 2).

We examined further the possibility that hepcidin secreted from C6 cells was responsible for this decrease in hBMVEC Fpn expression. With C6 cell total RNA as template, RT-PCR analysis demonstrated the presence of hepcidin mRNA in this glial cell line indicating that hepcidin is likely part of the C6 cell secretome (Fig. 7B). We also used the hepcidin antagonist, fursultiamine [41], as an indirect test that the knock-down in hBMVEC Fpn was due to the action of hepcidin. Fursultiamine binds to Fpn preventing the hepcidin-induced post-translational ubiquitination of Fpn. This drug does not hinder the efflux of iron from Fpn [41].

Indeed, a 24 h fursultiamine treatment resulted in complete inhibition of hBMVEC Fpn turn-over when C6 cells were seeded proximal to endothelial cells (Fig. 7A, bottom panels); quantitatively, the average fluorescent intensity associated with hBMVEC Fpn was restored with fursultiamine treatment in comparison to the no-drug control (Fig. 7C, closed bars). Furthermore, fursultiamine treatment restored ^59^Fe-efflux activity from hBMVEC in this co-culture orientation (Fig. 7D, compare open bars to closed bars). Fursultiamine did not stimulate hBMVEC ^59^Fe-efflux when hBMVEC were seeded alone, or seeded distal to C6 cells in transwell (Fig. 7D). These data are fully consistent with the interpretation that fursultiamine is blocking a hepcidin-dependent stimulation of hBMVEC Fpn turn-over. While the specificity of the commercial Fpn antibody used in these experiments has not been established elsewhere, the fact that the immunofluorescence is suppressed upon treatment with hepcidin (Fig. 6B), a decrease that is blocked by addition of a known hepcidin antagonist (Fig. 7A, C) is strong evidence that Fpn is the protein being imaged in these experiments.

### Proximal C6 cells induce hBMVEC Fpn ubiquitination and internalization

**Figure 8:**
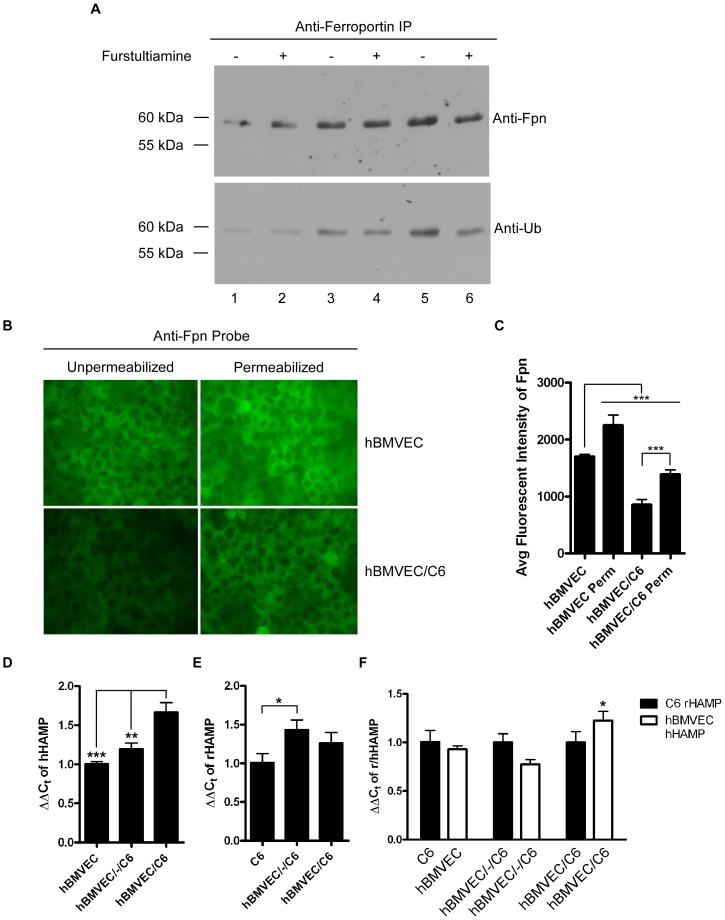
Proximal C6 cells induce the internalization and mono-ubiquitination of hBMVEC Fpn. (A) Fpn was immunoprecipitated from lysates of hBMVEC seeded in transwell either alone (lanes 1–2), distal to (lanes 3–4), or proximal to (lanes 5–6) C6 cells. Lysates from hBMVEC cultured under the same co-culture conditions but treated for 24 h with 10 µM fursultiamine were probed for Fpn as well. Fpn-immunoreactive eluates were probed by immunoblots for either Fpn or ubiquitin (Ub) conjugated to Fpn. (B) hBMVEC alone or seeded proximal to C6 cells were fixed and permeabilized (0.1% Tween-20) or not followed by processing for indirect immunofluorescence imaging of Fpn. The images were obtained with a 40X objective. (C) Average fluorescent intensity of hBMVEC Fpn from 4–7 separate fields of view from each condition obtained (from (B)) using the AxioVision software. (D) qPCR of human HAMP within total RNA isolated from hBMVEC seeded in transwell either alone (hBMVEC), distal to (hBMVEC/-/C6), or proximal to (hBMVEC/C6) C6 glioma cells. (E) qPCR of rat HAMP within total RNA isolated from C6 glioma cells grown in transwell either alone (C6), distal to (hBMVEC/-/C6), or proximal to (hBMVEC/C6) hBMVEC. (F) hBMVEC HAMP gene expression relative to C6 glioma cells in each of the three transwell orientations. All hBMVEC HAMP values are relative to C6 glioma HAMP in each orientation (C6 HAMP expression are set to 1 in each orientation). One-way ANOVA statistical analysis was used to determine significance. *P<0.05, **P<0.01, ***P<0.001. Data are represented as means ± S.D. (n = 3, technical replicates).

Hepcidin-induced Fpn internalization is initiated by the binding of hepcidin to Fpn. A subsequent ubiquitination of Fpn leads to the internalization of the protein [23], [24]. Our data indicated that C6 cell-secreted hepcidin likely induced hBMVEC Fpn internalization. We sought to investigate the presence of ubiquitinated Fpn in hBMVEC lysates grown in transwell in each orientation in the presence and absence of 10 µM fursultiamine. hBMVEC lysates were immunoprecipitated with anti-Fpn antibody and the immunoprecipitates were subsequently immunoblotted with anti-Fpn or an anti-ubiquitin antibody that recognizes both mono- and poly-ubiquitinated species (mAB clone FK2). This western analysis showed that the abundance of Fpn protein in hBMVEC grown in the three orientations (Fig. 8A, anti-Fpn). Ubiquitination of the protein was significantly enhanced in the proximal orientation, an increase that was suppressed by fursultiamine (Fig. 8A, anti-Ub; compare lane 6 to lane 5). We note that ubiquitinated-Fpn detected here in hBMVEC does not migrate as would be expected of a poly-ubiquitinated species.

Fpn ubiquitination triggers internalization and subsequent degradation in the lysosome [42]. Antibodies specific to extracellular epitopes on Fpn have been used to track the protein's internalization [25]; we used this specific immune-detection strategy to determine if hBMVEC Fpn was recycled from the plasma membrane when grown proximal to C6 cells. hBMVEC grown in the proximal orientation with respect to the glial cell line displayed significantly less surface-accessible Fpn as compared to hBMVEC grown alone (Figs. 8B and 8C); these differences were quantified by the average fluorescent intensity of hBMVEC Fpn visualized in unpermeabilized cells. Permeabilization demonstrated that the fraction of Fpn that was intracellular increased when C6 cells were grown in the proximal orientation. Approximately 32% of hBMVEC Fpn was intracellular when grown alone whereas ∼62% of Fpn was intracellular when hBMVEC were grown proximal to the glial cell line (Fig. 8C). These data suggest that Fpn was recycled from the plasma membrane in the presence of proximal C6 cells but had not undergone appreciable hepcidin-mediated degradation. We cannot, however, exclude the possibility that newly synthesized Fpn contributed to the cytoplasmic hBMVEC Fpn detected by indirect immunofluorescence of permeabilized cells.

### hBMVEC and C6 cell hepcidin transcript abundance is altered by proximity

The apparent increase in hepcidin-dependent hBMVEC Fpn internalization when grown proximal to C6 cells suggested the hypothesis that in this orientation, hBMVEC and/or C6 cell hepcidin expression was increased. A similar effect was demonstrated with human umbilical vein endothelial cells and hepatocytes grown in co-culture [43]. Thus, we collected total RNA from both hBMVEC and C6 cells seeded in three orientations, 1) alone, 2) seeded distal to one another, or 3) seeded proximal to one another. RNA was obtained after 5 days of co-culture growth and was analyzed for hepcidin transcript (HAMP) levels via qPCR. In each cell type, proximity appeared modulate regulation of HAMP gene expression (Figs. 8D–E) although the overall differences were not pronounced (Fig. 8F). The absence of a significant change in hepcidin expression suggests the hypothesis that it is the effective concentration of hepcidin that is modulated by cell-cell proximity, increasing at the hBMVEC as the glial cell end-feet grow spatially close to the basal membrane of the hBMVEC. Whether hepcidin from both cell types is required for the observed hepcidin-mediated hBMVEC Fpn down regulation was not addressed by our experimental design.

## Discussion

Change in the rate of mammalian brain iron accumulation throughout development has suggested that astrocytes play a key role in regulating the efflux of iron from BMVEC into the brain interstitium [44], [45]. Using a transwell, co-culture model of the BBB we have obtained evidence for the regulatory interactions between BMVEC and C6 cells that may be central to the mechanism underlying the established physiologic developmental pattern of brain iron accumulation. These observations are significant because in the absence of astrocytes, neurons may become susceptible to oxidative stress caused by iron flux into the brain; a mechanism in which iron influx is depressed by astrocytes would prevent this pathophysiologic condition. There is ample evidence that astrocytes play a major role in managing the flux of circulating metabolites from blood into the CNS [46]; we suggest that our work here has provided new details on how these glial cells might manage brain iron accumulation.

*In vivo* data have demonstrated that in the rat brain at P0, when astrocytes are not proximal to the basal surface of BMVEC, iron accumulates in these endothelial cells; a corresponding lack of iron transport into the brain parenchyma is observed [47], [48]. In the absence of C6 cells, hBMVEC display a base-line iron efflux, most of which is directed apically, towards the chamber containing serum. These data suggest that *in vivo* hBMVEC at P0 likely efflux the majority of their accumulated iron back into the circulation. Note that iron flux from BMVEC back into the blood flow was not examined in published experiments [47], [48]. Since hBMVEC Fpn may be expressed on both the apical and basal surface it is likely that the BBB acts as a conduit for both brain iron import and export [10]. Consistent with this inference, we have shown that both serum and C6 cells influence the polarity and extent of iron efflux from hBMVEC.

From P0 to P14, *in vivo* evidence indicates a dramatic influx of iron into the brain paired with an appreciable loss of iron from BMVEC [44], [47], [48]; astrocyte-endfeet extend into the local microenvironment surrounding BMVEC from P0 to P14 [8]. The increase in proximity of astrocytes to BMVEC from P0 to P14 allows for components of the astrocyte secretome to induce or suppress activity from BMVEC; an example of this is the induction of BMVEC tight-junction formation by astrocyte-conditioned media [49]. Our data indicate that astrocytes provide enhanced hBMVEC iron efflux through secretion of sCp which provides the ferroxidase activity necessary to export iron through hBMVEC Fpn; *in vitro*, sCp has been shown to catalyze an appreciable amount of ^59^Fe-efflux from hBMVEC Fpn [10]. Although hBMVEC endogenously express both Hp and sCp [10], in the absence of serum (-serum media) iron efflux is minimal. Therefore, *in vivo*, we propose that exogenous ferroxidase activity, provided by neighboring astrocytes in the form of sCp, is required to fully support hBMVEC iron efflux.

At P14, astrocyte endfeet have completed their ensheathment and are in close contact with the basal surface of BMVEC [8]. At this developmental juncture, there is a loss of BMVEC Fpn expression with a subsequent reduction in total brain iron-uptake [9], [44]. Our *in vitro* data indicate that this decline in brain iron-uptake may be a direct consequence of BMVEC Fpn depletion due to the peptide hormone hepcidin secreted by astrocytes at least in part; injected into mouse brain, hepcidin induces the turnover of Fpn [50]. In contrast, Hepc^−/−^ mice contain higher levels of Fpn protein in endothelial cells of the blood-retinal barrier than do wild type mice resulting in increased retinal iron accumulation with subsequent retinal degeneration [26]. These several observations correlate with the loss-of-function iron-efflux phenotype in hBMVEC observed in our *in vitro* model BBB system.

The stimulated internalization of Fpn due to hepcidin is concentration-dependent [24], [25], [51] and is modulated by the ferroxidase activity required for iron efflux provided by Hp and/or Cp. Thus, in the absence of a ferroxidase, Fpn is very sensitive to hepcidin, but is less so in the presence of this activity. We suggest that with C6 cells in a distal orientation relative to hBMVEC the hepcidin concentration at the hBMVEC is too limited to stimulate appreciable Fpn turnover, particularly under the condition in which hBMVEC are stimulating the expression and secretion of sCp by C6 cells. In contrast, when the C6 cells are proximally located to hBMVEC the spatial volume between the two cell types shrinks significantly; this allows for a critical concentration of hepcidin to be reached, thus inducing the turnover of hBMVEC Fpn. This model is analogous to neurotransmitter concentration in a synaptic cleft [52]. Indeed, calculations indicate that the 1 mM glutamate estimated in this cleft correlates with as few as 10^4^ molecules of glutamate or 10^−19^ mol [53].

Hepcidin induces the internalization and ubiquitination of Fpn [23], [24], [40]. Ubiquitination signals the Fpn-hepcidin complex to be trafficked to the lysosome where Fpn degradation occurs [42]. While we would have expected to observe poly-ubiquitinated species of Fpn in our system, we were able to detect only the mono-ubiquitinated form in our immunoblots. Fpn mono-ubiquitination has been observed previously [23]. Furthermore, while a significant amount of hBMVEC Fpn was recycled from the plasma membrane when grown proximal to astrocytes, only a fraction of this Fpn appeared to undergo hepcidin-mediated catabolism. This result could be due to synthesis of new Fpn, a lack of hepcidin mediated Fpn degradation, or some combination of the two. Irrespective of mechanism, hBMVEC Fpn was internalized and its function was lost from hBMVEC when the cells were grown in contact with the glia-derived cell line.

The hepcidin-Fpn interaction can be blocked pharmacologically *via* incubation with the thiamine derivative fursultiamine; blocking the binding of hepcidin to Fpn prevents Fpn internalization and subsequent ubiquitination [41]. In our BBB model system, fursultiamine prevented the loss of surface-expressed hBMVEC Fpn as well as the subsequent loss of hBMVEC iron efflux when C6 cells were seeded proximal to astrocytes (Fig. 7). Fursultiamine also reduced the ubiquitination of hBMVEC Fpn that occurred when hBMVEC were grown in this orientation (Fig. 8A). These results are consistent with the hypothesis that hepcidin was the paracrine agent responsible for hBMVEC Fpn internalization. Primary cell cultures derived from Hepc^−/−^ mice could be used to confirm this hypothesis. It should be noted however, that fursultiamine did not reduce Fpn ubiquitination when hBMVEC were seeded distal to C6 glioma cells (hBMVEC/-/C6) (Fig. 8A). hBMVEC Fpn, grown in this orientation, may be undergoing hepcidin-independent ubiquitination. Hepcidin-independent ubiquitination of Fpn has been demonstrated to occur under circumstances of low intracellular iron [54]. hBMVEC iron efflux is at its greatest when grown distal to C6 glioma cells, potentially leading to a large enough decrease in intracellular iron that endogenous Fpn is becoming ubiquitinated independent of exogenous hepcidin. Fursultiamine has not been demonstrated to inhibit hepcidin-independent ubiquitination of Fpn.

Recently, a ferroxidase activity homologous to that seen in FHC has been localized to the E2 domain of APP [14], [55], although more recent data have challenged this inference [16], [17]. The cleavage product of APP, amyloid-beta peptide (Aβ), has been linked to the disease model of Alzheimer's disease [37], [56]. Alzheimer's disease is marked by an accumulation of Aβ (amyloid plaques) in the brains of patients. Aβ accumulation is first seen in the vicinity of BMVEC (cerebral amyloid angiopathy) where it appears to induce toxic effects such as oxidative stress, ion channel dysfunction, inflammation, and apoptosis in both BMVEC and astrocytes [37], [57], [58]. Both BMVEC and astrocytes express APP [19], [21]. We demonstrated that the FD1 peptide containing the putative ferroxidase active site of the E2 domain of APP [14], [16] stimulated hBMVEC ^59^Fe-efflux (Table 1). Cleavage of APP in the vicinity of BMVEC could increase sAPP in the cleft between BMVEC and astrocytes, effectively suppressing the hepcidin-induced internalization of BMVEC Fpn. This, in turn, could increase the uptake of brain iron across the BBB thus accelerating the progression of Alzheimer's disease; brain iron accumulation is a characteristic of this disease [59]–[62]. The insights gained from this and prior studies may provide a gateway for identifying potential drug targets aimed to slow, prevent, or reverse excessive brain iron accumulation.

## Materials and Methods

### Cell culture and reagents

hBMVEC were obtained from Dr. Supriya Mahajan (University at Buffalo); the generation and characteristics of this cell line have been described in detail [63]. HepG2, HEK293T, Caco-2, rat C6 glioma, and primary rat astrocytes were obtained from Cell Applications (San Diego, CA). These cell lines were cultured in RPMI 1640 as previously described [64]. Experiments were performed in 24-well tissue-culture dishes or Greiner bio-one transwells (1.0 µm pore size) as specified. Rat bi-potential oligodendrocyte-type 2-astrocyte (O-2A) progenitor cells (CG4 cells) were provided by Dr. Fraser Sim (University at Buffalo); this established cell line has been described in detail [65] and were cultured in M41 media (DMEM/F12/N1) plus growth factors PDGF (10 ng/mL) and FGF (10 ng/mL) from Peprotech (Rocky Hill, NJ). Primary rat hippocampal neurons were cultured in Neurobasal media (Life Technologies, Carlsbad, CA) with 0.5 mM Glutamine. These cells were a gift from Ms. Changyi Ji; they were obtained from fetal rat brains under a protocol approved by the IACUC of the University at Buffalo to Dr. Daniel Kosman, Approval Number BCH29101N. FD1 peptide (sequence; HRERMSQVMREWEEAERQAKNL) was synthesized by Genscript (Piscataway, NJ). Human hepcidin-25 peptide (ab31875) was purchased from Abcam (Cambridge, MA). Recombinant forms of light and heavy chain ferritin subunits (L/H-ferritin) were a gift from Dr. Richard Watt (Brigham Young University).

### Indirect immunofluorescence

hBMVEC were fixed for 10 minutes with 3.7% paraformaldehyde and 4% sucrose in PBS, incubated for 30 minutes in PBS 1% BSA and 0.3 M glycine, for 30 minutes with anti-SLC40A1 (Abcam ab85370, 1∶100 dilution) in PBS 1% BSA, then for 30 minutes with Alexa 488-conjugated goat anti-rabbit (1∶1000, Invitrogen) in PBS 1% BSA. Anti-SLC40A1 (Abcam ab85370, 1∶100 dilution) was used for preparations; anti-SLC40A1 antibody ab85370 targets a Fpn epitope predicted to be extracellular [66]. Coverslips were mounted onto glass slides using SlowFade gold antifade reagent with DAPI (Life Technologies). Where applicable, cells were permeabilized during blocking with 0.1% Tween 20. Images were obtained using a Zeiss AxioImager Z1 Axiophot wide-field fluorescence microscope and were analyzed by Zeiss AxioVision software. As noted in the figure legends, both 20X and 40X magnification were used.

### ^59^Fe efflux assays

hBMVEC were loaded with ^59^Fe^II^-citrate using a reductase-independent uptake protocol based on the presence of 5 mM dihydroascorbate and 250 µM citrate (± serum). Alternatively, cells were loaded with ^59^Fe-transferrin (^59^TBI) plus 250 µM citrate. Reactions were quenched with ice-cold quench buffer as previously described [64], and lysed with RIPA buffer (Sigma-Aldrich, St. Louis, MO). Lysates were assayed for ^59^Fe and protein concentration. For efflux assays, hBMVEC were loaded for 24 h, washed with serum-free RPMI 1640 containing 250 µM citrate, and incubated with appropriate reagents (sCp, conditioned-media, etc.) ± serum for 0 h, 24 h, or 48 h. The media was collected and the cells were quenched and processed as described above. For transwells, aliquots of media from upper and lower chambers were taken at each time point. Cell-associated ^59^Fe values (LKB Wallac CompuGamma) were normalized by protein concentration.

### Transwell model system

C6 glioma, HEK293T, or HepG2 cells (0.15×10^5^ cells) were seeded either on the bottom of the lower chamber or on the undersurface of the transwell membrane while 0.15×10^5^ hBMVEC were seeded onto the upper-surface of the transwell membrane; the cultures were incubated for 5 days. TEER values were taken using a Millicell ERS-2 Epithelial Volt-Ohm Meter according to the manufacturer's instructions (EMD Millipore, Billerica, MA). Indirect immunofluorescence imaging of the hBMVEC on the membrane was performed as described for monolayers.

### Ferroxidase activity assay

Ferroxidase activity assays were performed as previously described with modifications [10]. Conditioned media (30 mL) was collected, filter sterilized, and concentrated to 100 µL using a 10,000 MWCO centrifugal filter (Millipore, Billerica, MA). Assays were performed in 100 mM MES buffer pH 6.0 using 10 µM freshly prepared ferrous ammonium sulfate and 80 µL of media concentrate. Ferrozine (100 µM) was used as the colorimetric indicator of ferrous iron producing an absorbance at 550 nm. All assays were blanked for iron-only controls.

### Immunodepletion and immunoblots

Immunodepletion of sCp from CCM was carried out using the Pierce Classic IP Kit (Thermo Scientific, Pittsburgh, PA) as per the manufacturer's instructions with modifications. Briefly, concentrated (300×) CCM was pre-cleared and applied to a column containing protein A/G plus agarose resin in which affinity purified anti-Cp antibody (Bethyl Laboratories, Cat. No. A80-124A, Montgomery, TX) had been immobilized (+ anti-Cp) or no antibody (- anti-Cp; control) had been immobilized. This CCM, antibody, agarose slurry was incubated overnight at 4°C with end-over-end rotation. The CCM flow-through was collected and applied to various assays as described. Bound Cp was eluted from the columns using the low-pH elution buffer provided. Immunoprecipitation of Fpn from hBMVEC lysates was performed using the Pierce Classic IP Kit as per the manufacturer's instructions. Equal amount of protein from each lysate was incubated with Fpn antibody and loaded onto separate columns.

Immunoblots were performed as previously described with minor changes [10]. Briefly, 30 mL CCM was concentrated (300×) and 20 µL of that concentrate was separated *via* SDS-PAGE (8%). The membrane was blocked 1 h with 5% BSA in PBS at 4°C, incubated overnight at 4°C with goat anti-Cp antibody (1∶10,000 dilution), followed by incubation with secondary anti-HRP antibody (Santa Cruz Biotechnology, Santa Cruz, CA). The eluted samples from IP of Fpn were separated as described and assayed for Fpn (ab85370) or ubiquitin (FK2 antibody; Enzo Life Sciences, BML-PW8810, Farmingdale, NY). Fpn and FK2 primary antibodies were detected using the Clean-Blot IP Detection Reagent (HRP) (Thermo Scientific, Pittsburgh, PA) as per the manufacturer's instructions.

### RT-qPCR

Total RNA was extracted from hBMVEC and C6 glioma cells using the TRIzol reagent (Invitrogen, Carlsbad, CA) as per the manufacturer's instructions. After DNAse treatment, RNA was reverse-transcribed using the SuperScript III Reverse Transcriptase (Invitrogen) with gene-specific (HAMP, Fpn, or sCp and β-actin) primers as per the manufacturer's instructions. SSoAdvanced SYBR Green Supermix (Bio-Rad, Hercules, CA) was used for the amplification and detection of cDNA. PCR reactions were performed using the Bio-rad CFX-96 real-time PCR instrument (Bio-Rad). In all cases β-actin was used as an internal control. Endpoint qPCR reactions were separated on a 1.7% agarose gel to confirm product size. Primers used are listed in Table S1.

### Other Procedures

To make conditioned media, cells were grown to confluency, washed, incubated overnight in their respective serum-free media, and filter-sterilized prior to use. Fursultiamine (10 µM; AK Scientific, Inc., Union City, CA) was added to culture media for 24 h. Protein concentrations were determined using the Pierce BCA protein assay (Thermo Scientific) as per the manufacturer's instructions. Apo-Tf (Sigma-Aldrich) was loaded with ^59^Fe as previously described [64].

### Statistical Analysis

All statistical analyses were performed by using Prism 5.0 (GraphPad Software, San Diego, CA). Paired t-tests were used in Table 1 as comparisons were made between two conditions (one variable) from the same time point. Comparisons of multiple samples were made by One-way ANOVA statistical analyses.

## Supporting Information

Figure S1 **Transwell ^59^Fe distribution after 24 h loading period.** hBMVEC were seeded in transwell either alone (hBMVEC), distal to (hBMVEC/-/C6), or proximal to (hBMVEC/C6) C6 glioma cells. Cells were grown for 5 days after which media was switched to RPMI 1640 with serum (apical) or without serum (basal). ^59^NTBI was added to the upper chamber of the transwells for 24 h. After 24 h loading period, media was taken from the upper and lower chambers and the total counts per minute (C.P.M.) were obtained. Data are represented as means ± S.D. (n = 3, technical replicates).
(TIF)

Figure S2 **hBMVEC Fpn gene expression is not altered by C6 glioma cell proximity.** hBMVEC were grown in transwell either alone (hBMVEC), distal to (hBMVEC/-/C6), or proximal to (hBMVEC/C6) C6 glioma cells. After 5 days, total RNA was isolated from hBMVEC and qPCR was performed to assess the relative levels of Fpn transcript. Data are represented as means ± S.D. (n = 3, technical replicates).
(TIF)

Table S1 **List of primers used for RNA analysis.**
(DOCX)
