# Supplementary figures and images for: Glial Cell Ceruloplasmin and Hepcidin Differentially Regulate Iron Efflux from Brain Microvascular Endothelial Cells
Source: PLoS One. 2014 Feb 12;9(2):e89003. doi: 10.1371/journal.pone.0089003 (PMC3923066; doi:10.1371/journal.pone.0089003)

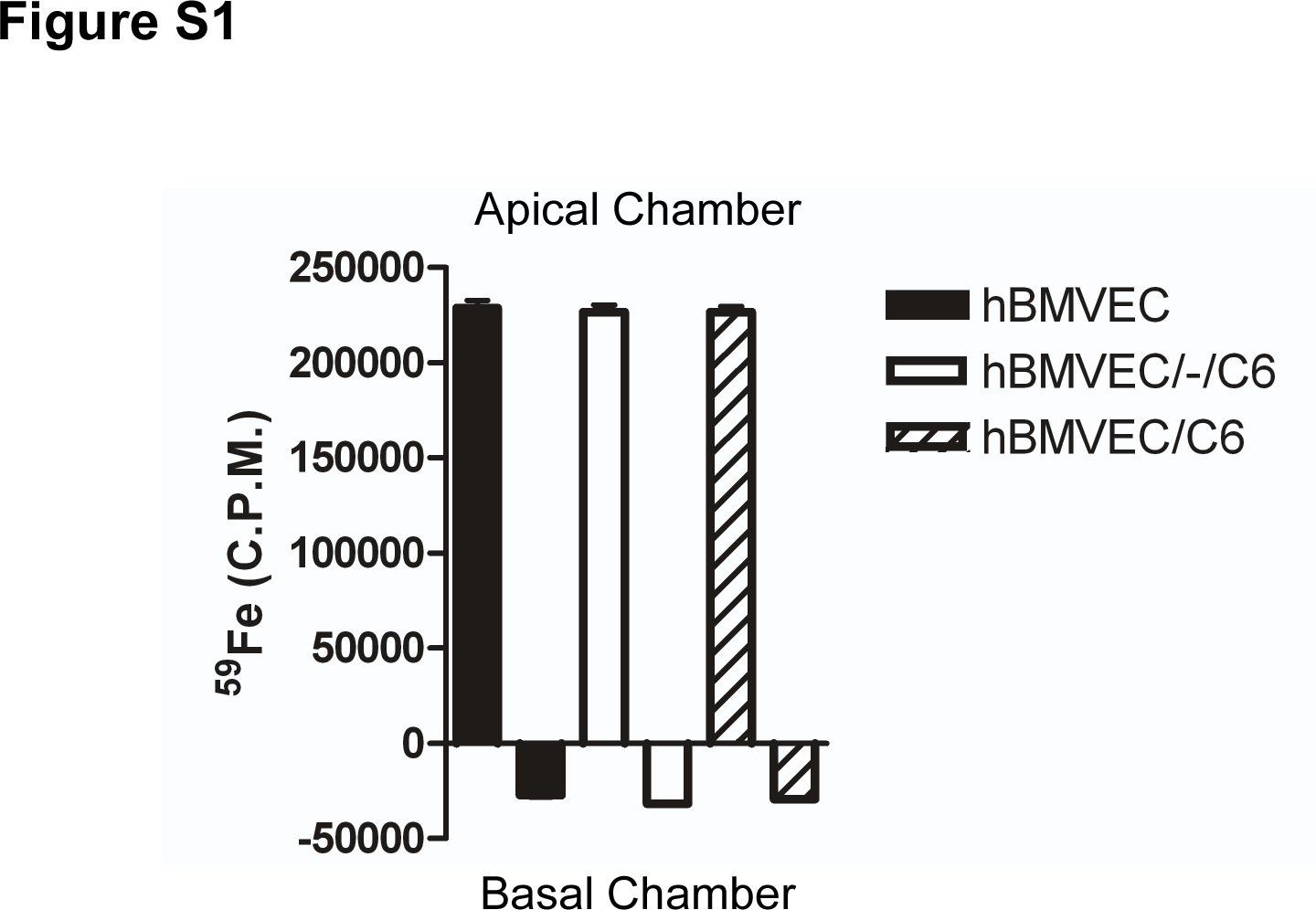

Supplement: Figure S1 — Transwell 59Fe distribution after 24 h loading period. hBMVEC were seeded in transwell either alone (hBMVEC), distal to (hBMVEC/-/C6), or proximal to (hBMVEC/C6) C6 glioma cells. Cells were grown for 5 days after which media was switched to RPMI 1640 with serum (apical) or without serum (basal). 59NTBI was added to the upper chamber of the transwells for 24 h. After 24 h loading period, media was taken from the upper and lower chambers and the total counts per minute (C.P.M.) were obtained. Data are represented as means ± S.D. (n = 3, technical replicates). (TIF) [file pone.0089003.s001.tif]

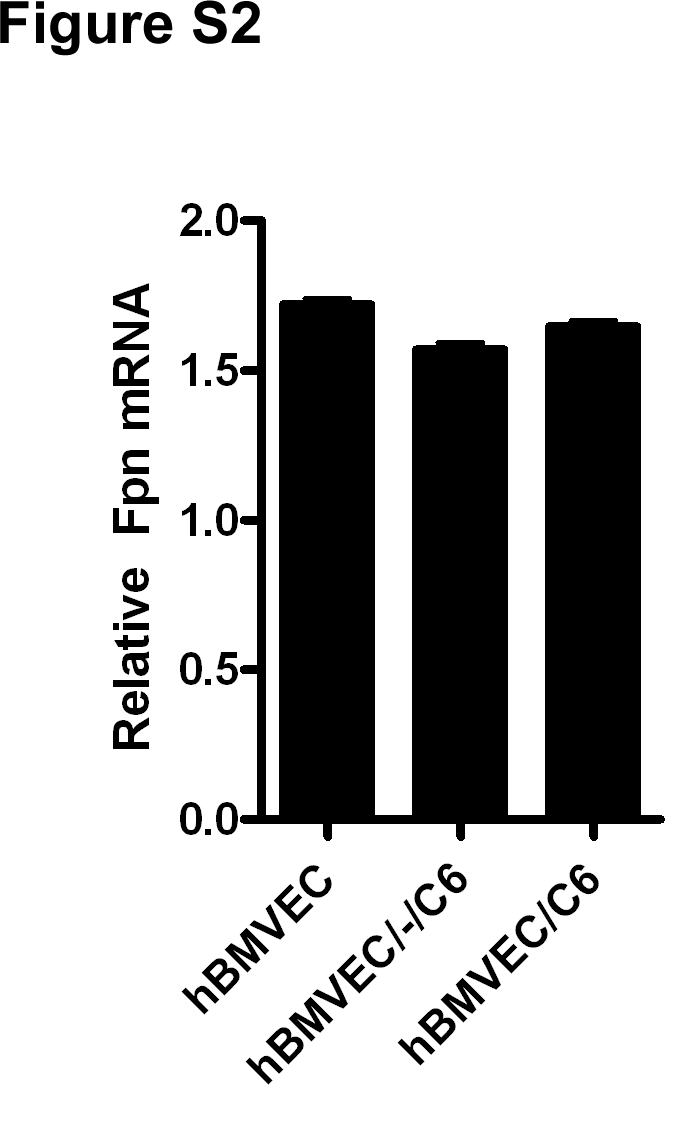

Supplement: Figure S2 — hBMVEC Fpn gene expression is not altered by C6 glioma cell proximity. hBMVEC were grown in transwell either alone (hBMVEC), distal to (hBMVEC/-/C6), or proximal to (hBMVEC/C6) C6 glioma cells. After 5 days, total RNA was isolated from hBMVEC and qPCR was performed to assess the relative levels of Fpn transcript. Data are represented as means ± S.D. (n = 3, technical replicates). (TIF) [file pone.0089003.s002.tif]
