# Supplementary material for: Glial Cell Ceruloplasmin and Hepcidin Differentially Regulate Iron Efflux from Brain Microvascular Endothelial Cells
Source: PLoS One. 2014 Feb 12;9(2):e89003. doi: 10.1371/journal.pone.0089003 (PMC3923066; doi:10.1371/journal.pone.0089003)
Supplement: Table S1 — List of primers used for RNA analysis. (DOCX) [file pone.0089003.s003.docx]

| Table 1. Primer list used for RNA analysis | | |
| --- | --- | --- |
| Transcript | **Forward Primer** | **Reverse Primer** |
| Β-actin | GGGTCAGAAGGACTCCTACG | GGTCTCAAACATGATCTGGG |
| Secreted (soluble) Rat Ceruloplasmin | TCCACTGCCATGTGACTGAC | TCGGCATTACCAATTCCCTCA |
| Rat Hepcidin (rHAMP) | GAAGGCAAGATGGCACTAAGCA | TCTCGTCTGTTGCCGGAGATAG |
| Human Hepcidin (hHAMP) | ACAGACGGGACAACTTGCAG | CTCCTTCGCCTCTGGAACAT |
| Human Fpn | CAGGGACTGAGTGGTTCCAT | GGAGATTATGGGGACGGATT |
